# Red blood cell distribution width (RDW) as a predictor of multiple organ dysfunction in pediatric critical care: a retrospective study

**DOI:** 10.1186/s12887-025-06294-0

**Published:** 2025-12-29

**Authors:** Syed M. Dayyan Hassan, Abdul Hadi Shahid, Zuhaib Ali, Muneeb Ahmed, Hasheem Mohammad, Syeda Farwa Fatima, Shalni Golani, Fatima Jamshaid, Najeeb Rahman, Naveed ur Rehman Siddiqui

**Affiliations:** 1 Medical College, Aga Khan University, Karachi, 74800 Pakistan; 2 Wayne State University, Trinity Health, Livonia, MI 48154 USA; 3 Department of Pediatrics, Aga Khan University, Karachi, 74800 Pakistan; 4 Department of Pediatrics and Child Health, Medical College, Aga Khan University, Karachi, 74800 Pakistan

**Keywords:** RBC distribution width, RDW, MODS, PICU, Length of stay, Mortality

## Abstract

**Background:**

RBC distribution width is a key variable in complete blood counts, associated with immature RBC release into circulation due to various processes, including systemic inflammation. RDW correlates with elevated acute inflammatory markers like ESR, CRP, and interleukin-6, and is a biomarker in conditions like kidney disease and multiple myelomas. It independently predicts disease severity in critically ill adults and is associated with morbidity, mortality and length of stay in pediatric intensive care unit, though its potential as an early biomarker for detecting pediatric patients with multiple organ dysfunction (MODS) remains unknown.

**Methods:**

The study retrospectively reviewed PICU patients admitted to Aga Khan University Hospital from September 2018 to December 2022, excluding those admitted for less than 48 h for elective procedures, received recent RBC transfusions, or were anemic. RDW > 14.0% was considered elevated. MODS, defined as dysfunction in two or more organs, was the primary outcome. Data included demographics, PRISM III scores, laboratory values (RDW, BUN, creatinine, CRP, etc.), and clinical outcomes. Patients were stratified into three RDW groups: <13.4%, 13.4–14.3%, and > 14.4%. Analysis focused on associations between RDW levels and MODS within the first 7 days of PICU admission.

**Results:**

The study included 680 patients. Higher RDW was associated with younger age and higher PRISM III scores, but not with sex. RDW Group III had longer hospital stays, higher mortality, and higher incidence of MODS, but not significant. Hemoglobin and MCHC levels were lower in Group III, whereas BUN and creatinine levels showed no significant differences across groups. The OR for MODS was highest for Group II.

**Conclusions:**

This retrospective study evaluated the prognostic value of RDW in predicting length of stay, mortality, and early identification of MODS within seven days. Among 680 pediatric patients, higher RDW levels were associated with increased mortality, longer LOS, and higher rates of sepsis and MODS, though these findings lacked statistical significance. Elevated RDW was linked to inflammation and critical illness severity but did not correlate well with pediatric severity scores or MODS trends. Future multicenter studies are recommended to explore RDW’s utility in predicting early organ dysfunction and critical illness outcomes.

## Introduction

RBC distribution width (RDW) is one of several variables routinely reported on a standard complete blood count (CBC) laboratory test [1]. An increase in RDW is observed in processes that cause the release of immature RBCs into circulation [2]. One such process is the systemic inflammatory response in critical illness, which impairs iron metabolism and inhibits the production of and response to erythropoietin, thereby impacting erythropoiesis and RBC maturation [3].

Although the precise mechanism remains unclear, data suggests that RDW may be associated with acute inflammatory states. This is evidenced by the correlation between RDW and elevated acute phase reactants such as erythrocyte sedimentation rate (ESR), C-Reactive Protein (CRP), and interleukin- 6 in both the general adult population and heart failure patients [4]. RDW has also been identified as an inflammatory biomarker in various other conditions, such as acute and chronic kidney disease, as well as multiple myelomas [4]. Additionally, it has been demonstrated to be an effective and independent predictor of the severity of the disease and adverse outcomes in critically ill adult patients [5, 6].

In two recent pediatric studies, RDW was found to be associated with morbidity, mortality, and length of stay (LOS) in the pediatric intensive care unit (PICU) [7, 8]. However, RDW’s potential as an early biomarker for detecting children with multiple organ dysfunction syndrome (MODS) is currently unknown. The effectiveness of RDW in predicting disease severity and its prognosis in critically ill pediatric patients, especially with regards to MODS, is understudied.

In this study, we aim to investigate RDW’s potential as an early biomarker for MODS in pediatric patients. By comparing RDW values with other laboratory markers and clinical parameters, our study seeks to determine whether RDW could serve as a reliable indicator for early identification of high-risk pediatric patients. Since MODS is a major cause of hospital resource utilization, prolonged hospital stays, and mortality, a biomarker that can identify high- risk children should aid in timely identification, prompt intervention and effective resource utilization in pediatric critical care in resource limited settings.

## Methods

### Study design

This was a single center, retrospective review of all patients admitted between September 1, 2018, and December 31, 2022, to the PICU at Aga Khan University Hospital, an academic, tertiary care hospital. Data was collected after approval from Ethical Review Committee (ERC) of The Aga Khan University.

### Study population

The study included all patients admitted to PICU during the study duration. However, we excluded the patients who were admitted for less than 48 h for elective procedures, received packed RBC transfusion in the previous 120 days or were identified to be anemic.

### Sampling technique

A convenience sampling technique was employed, utilizing readily available data from the years 2018 to 2022. Following ethical approval from the ERC, data collection commenced in 2024, granting access to relevant medical records for the duration of the study.

### Data collection

The reference range for RDW is 11.5–14.0% and is not age-dependent so, RDW greater than 14.0% was considered increased [9]. The first CBC at admission was taken into account for RDW. The primary outcome was the MODS, and the secondary outcomes included PICU LOS, hospital LOS, duration of Mechanical Ventilation, and mortality. MODS was defined as the presence of dysfunction of two or more organs and classified according to the 2005 International Consensus Conference on Pediatric Sepsis [10].

Admission severity of illness i.e. Pediatric Risk of Mortality (PRISM) III was calculated within 24 h of PICU admission using age specific clinical parameters like lowest systolic blood pressures, Heart rate, temperature, pupillary reflexes, mental status, acid base balance, chemical and hematological laboratory parameters as described in a previous study [11].

On review of each patient’s medical record, subjects whose clinical course met criteria for organ dysfunction of two or more organs at admission and/or during the first 7 days of PICU course was categorized as MODS by the investigators.

Demographic characteristics including age, sex, height, weight, admission diagnostic categories, admission date, and admission severity of illness using the PRISM-III were extracted from the medical records. We also obtained each subject’s Pack RBC transfusion history to exclude the patients who received it in the last 4 months from any medical health facility. Laboratory data was collected including a CBC obtained within 24 h of PICU admission from which the RDW was recorded. Furthermore, blood urea nitrogen (BUN) and creatinine were also recorded, if obtained within 24 h of PICU admission, as surrogate markers of renal status. Serum albumin was used as a surrogate for nutritional status and ESR, CRP, and Procalcitonin were recorded as markers of inflammation. The reference range used for RDW was 11.5–14.0% and was not age-dependent. The population was stratified into three groups using admission RDW values based on previously published a priori cut-off points; into group I (RDW < 13.4%), group II (13.4–14.3%), and group III (>14.4%) [7].

We defined anemia according to the World Health Organization definition: a hemoglobin lower than 11 g/dl for patients less than 5 years, lower than 11.5 g/dl for patients 5–12 years, lower than 12 g/dl for male patients 12 to less than 15 years and female patients 12 years old and older, and lower than 13 g/dl for male patients 15 years old and older [12]. All study data was collected on an Microsoft Excel Workbook and was stored in password protected folder at Aga Khan University Hospital desktop. Stata 17 software was used for further analysis and all files were kept under password protection.

### Data analysis

Frequencies and percentages were calculated for categorical variables and were compared over different outcome categories using chi-square test. Continuous variables were presented as mean ± SD or median with interquartile range as appropriate after testing for normality. Continuous variables were compared across different outcome categories using t-test and Mann Whitney test as appropriate. Logistic regression analysis was performed using RDW Group I as the reference group, with odds ratios (OR) and 95% confidence intervals (CI) calculated. A parallel analysis with mixed models was also used due to readmissions.

## Results

**Fig. 1:**
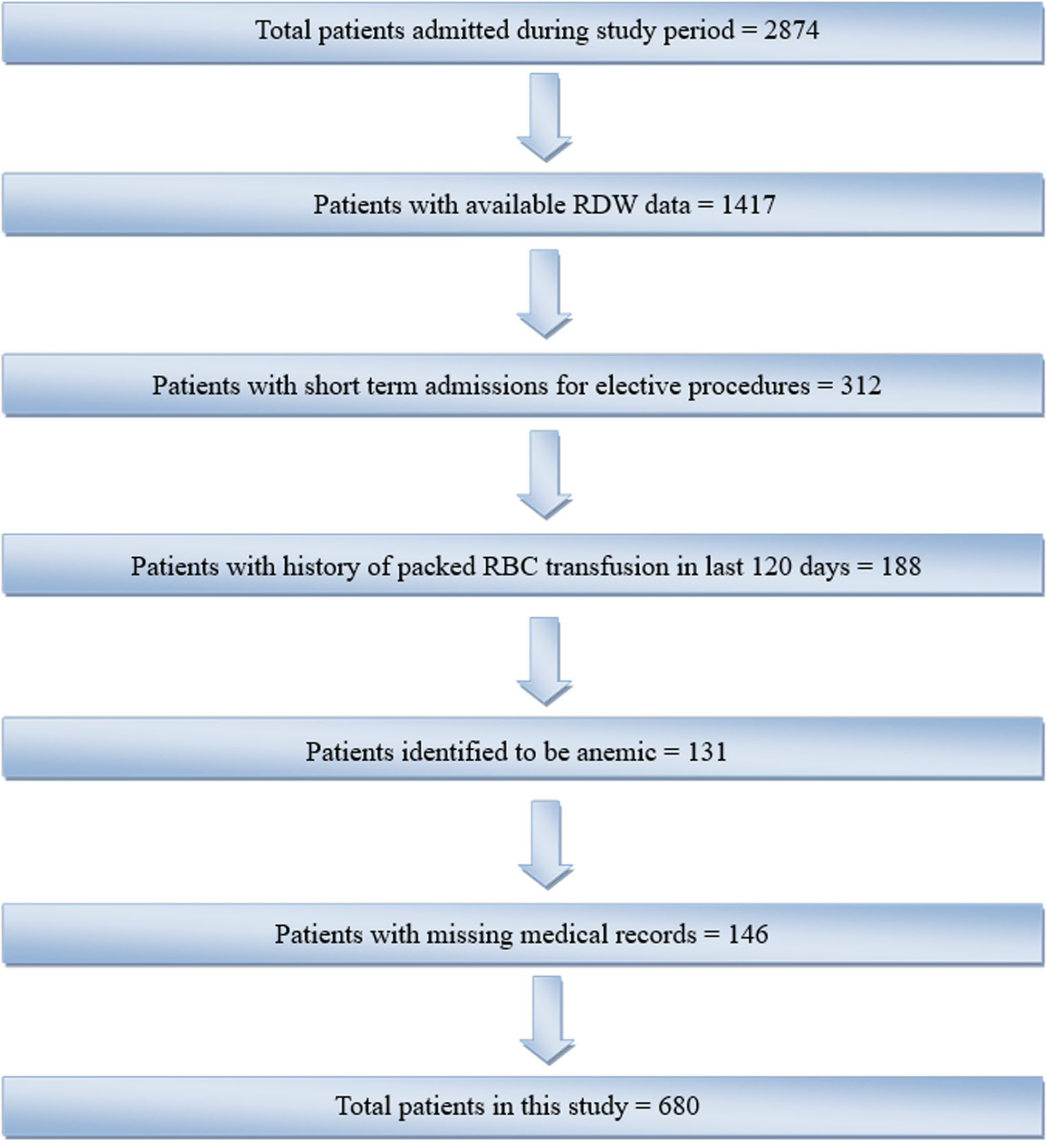
Schematic of the dataset used for the study (Study duration = September 1, 2018 to December 31, 2022)

During the period of study, 2874 patients were admitted to the PICU, out of which the data for RDW was available for 1417 patients which were admitted September 2020 onwards. However, 312 patients were admitted for routine short-term 48 h of postoperative care and around 188 patients had a history of packed RBC (PRBC) transfusion in the 120 days prior to PICU admission [8]. In addition to this, around 146 patients had missing medical records, and 131 patients were excluded because they were identified to be anemic (Fig. 1).

The study included a total of 680 pediatric subjects out of which 424(62.4%) were male and 256(37.6%) were female. While 97.9% of patients in study were admitted in PICU for first time, around 2.1% patients were readmitted later during the study period. The subjects were categorized into three groups based on their admission RDW levels: Group I (*N* = 85), Group II (*N* = 83), and Group III (*N* = 512) (Table 1).

**Table 1:** Demographics and clinical characteristics of cohort

	Total (*N* = 680)	Admission RDW Group			*p*-value^1^
		I ***(n = 85)***	II ***(n = 83)***	III ***(n = 512)***	
RDW	14.8 (13.4–17.1)	12.9 (12.7–13.2)	13.8 (13.6–14.0)	16.6 (15.2–18.9)	< 0.001
Age (in years)	3.4 (0.9–10.0)	6.0 (2.0–12.0)	6.0 (0.8–10.0)	3.0 (0.8–9.0.8.0)	0.001
Sex					0.85
Female	256 (37.6%)	33 (38.8%)	29 (34.9%)	194 (37.9%)	
Male	424 (62.4%)	52 (61.2%)	54 (65.1%)	318 (62.1%)	
PRISM III	4.3 (3.3–5.0.3.0)	4.3 (3.0–5.0)	4.5 (3.7–5.0.7.0)	4.3 (3.0–5.0)	0.43
Admit category					0.47
Respiratory	198 (29.1%)	22 (26.5%)	24 (29.2%)	152 (29.5%)	
Sepsis/Infection	127 (18.7%)	22 (26.5%)	7 (8.3%)	96 (18.7%)	
Neurological	76 (11.2%)	15 (17.6%)	17 (20.8%)	46 (8.8%)	
GI and Liver	72 (10.5%)	5 (5.9%)	3 (4.2%)	64 (12.4%)	
Haem/Oncology	67 (9.9%)	13 (14.7%)	4 (4.6%)	50 (9.8%)	
Metabolic/Endocrine	46 (6.8%)	3 (2.9%)	10 (12.5%)	34 (6.7%)	
Cardiovascular	42 (6.2%)	0 (0%)	10 (12.5%)	32 (6.2%)	
Musculoskeletal	11 (1.6%)	3 (2.9%)	0 (0%)	8 (1.6%)	
Genitourinary	8 (1.2%)	0 (0%)	3 (4.2%)	5 (1%)	
Miscellaneous	33 (4.4%)	3 (2.9%)	3 (4.2%)	27 (4.7%)	

^1^Chi-square test

The median age of patients varied across the groups, with the youngest median age observed in Group III (3.0 [0.8–9.0.8.0] years), followed by Group II (6.0 [0.8–10.0] years) and Group I (6.0 [2.0–12.0] years) with a p-value of 0.001, showing a significant trend that younger patients were more likely to present with higher RDW levels. However, there was no significant difference in the sex distribution among the groups (*p* = 0.86), indicating that sex did not play a role in the distribution of RDW levels (Table 1).

The median PRISM III score of entire population was 4.3 [3.3–5.0.3.0], and Group II had a higher PRISM III score of 4.5 [3.7–5.0.7.0]. However, there was no significant difference between these scores among different groups (*p* = 0.63). The admission category of patients in the study revealed the majority being admitted for problems related to the Respiratory system (29.1%). Moreover, 18.7% were admitted with category of sepsis/infection while 11.2% were admitted due to Hematology/Oncology disorders. In RDW group I, most subjects were admitted under category for Sepsis/Infection (26.5%) and Respiratory (26.5%). However, in group II admission categories for Respiratory (29.2%) and Neurological (20.8%) were highest and in Group III the admission categories for Respiratory (29.5%) and Sepsis/Infection (18.7%) were highest (Table 1).

Table 2 shows that the mean hemoglobin level was higher in Group I (13.0 ± 1.1) compared to Group I and Group II, in consistence to these findings, the average Mean Corpuscular Volume (MCV) was 83.0 ± 4.1 fl. in Group I compared to Group II (79.3 ± 5.2 fl.) and Group III (80.0 ± 9.3 fl.). Moreover, average Mean Corpuscular Hemoglobin Concentration (MCHC) for the cohort was 32.3 ± 1.5 g/dL and was significantly lower for Group III (31.9 ± 1.5 g/dL) with a p-value of 0.038. Additionally, blood urea nitrogen (BUN) and Creatinine levels were reported with no significant differences among the RDW groups (*p* = 0.82 and *p* = 0.67 respectively). These findings suggest that while RDW is associated with certain clinical parameters like MCHC, it does not necessarily correlate with others such as BUN and creatinine in predicting MODS in this pediatric population.

**Table 2:** Laboratory parameters and clinical characteristics of the patients

Parameters	Subjects (*N* = 680)	Admission RDW Group			*p*-value^1^
		I (*n* = 85)	II (*n* = 83)	III (*n* = 512)	
Hemoglobin	12.6 ± 1.7	13.0 ± 1.1	12.3 ± 1.4	12.4 ± 2.0	0.50
MCHC^2^	32.3 ± 1.5	33.1 ± 1.4	32.5 ± 1.3	31.9 ± 1.5	0.038
MCV^3^	80.7 ± 7.8	83.0 ± 4.1	79.3 ± 5.2	80.0 ± 9.3	0.41
Platelets	345 ± 218	328 ± 152	323 ± 268	358 ± 233	0.86
BUN^4^	11 (7–17)	10 (9–15)	10 (7–17)	12 (7–19)	0.82
Creatinine	0.4 (0.3–0.7)	0.5 (0.4–0.8)	0.4 (0.3–0.8)	0.4 (0.3–0.7)	0.67
CRP^5^	47.4 ± 55.8	82.4 ± 82.9	61.2 ± 79.5	39.3 ± 43.6	0.20
Procalcitonin	16.3 ± 30.6	10.8 ± 17.4	6.5 ± 12.8	17.9 ± 32.8	0.59
Length Of Stay	4.5 ± 4.6	3.9 ± 3.2	3.9 ± 3.5	4.6 ± 4.9	0.50
Status					0.55
Alive	586 (86.2%)	76 (89.7%)	75 (90.6%)	435 (84.9%)	
Expired	94 (13.8%)	9 (10.3%)	8 (9.4%)	77 (15.1%)	
MODS^6^	399 (58.7%)	45 (52.9%)	43 (51.8%)	311 (60.7%)	0.16

^1^Chi-square test

^2^Mean Corpuscular Hemoglobin Concentration in g/dL

^3^Mean Corpuscular Volume in fl

^4^Blood Urea Nitrogen in mg/dL

^5^C-Reactive Protein in mg/L

^6^Multiple Organ Dysfunction Syndrome

The mean Length-of-stay (LOS) among the entire population of study was 4.5 ± 4.6 days, with a higher LOS among the Group III (4.6 ± 4.9 days) than Group I and II. The status of patients at the end of their stay indicated that 86.2% were alive among the entire study population. However, the percentage of expired patients was highest in Group III (15.1%).

Table 2 also shows 58.7% of patients diagnosed with MODS. While it was higher in RDW Group III (60.7%) compared to Groups I and II, however it was not significantly different (*p* = 0.16). Table 3 shows the Odds Ratio (OR) of MODS associated with RDW groups which reveals an OR = 1.05 [0.57–1.92] for Group II, and OR = 0.73 [0.46–1.15] for Group III.

**Table 3:** Odds ratio (OR) of multiple organ dysfunction associated with admission RDW values

Admission RDW Group	OR (95% CI)	*p*-value^1^
I	1.000 (—)	
II	1.05 (0.57–1.92)	0.88
III	0.73 (0.46–1.15)	0.18

^1^Chi-square test

## Discussion

RDW is a widely available, cost-effective, and routinely included parameter in the complete blood count panel that reflects the size variability of RBCs in an individual [13]. Elevated RDW levels are indicative of increased variability in erythrocyte size, often associated with inflammatory conditions, oxidative stress, and impaired erythropoiesis [5, 13]. Due to its non-specific nature, elevated RDW has been linked to various disease processes, including cardiovascular, renal, chronic pulmonary diseases, and critical illness, suggesting its utility as a prognostic biomarker for morbidity and mortality in these conditions [14]. Our retrospective study aimed to evaluate the prognostic value of RDW levels at admission regarding PICU LOS, morbidity, mortality, and the association of elevated RDW levels with early identification of MODS within the first seven days of PICU admission.

The current study included 680 pediatric patients whose RDW levels were measured upon PICU admission at AKUH over five years. Comparable to previous studies, our study had a significant sample size that provided adequate statistical power to evaluate these associations [7]. The gender distribution across RDW groups in our study showed no significant differences, aligning with previous literature that reported no significant relationship between gender and RDW levels. A notable observation in our study was the significant trend showing younger pediatric patients more likely to have higher RDW levels, contrasting with earlier studies suggesting older age as associated with lower RDW values [15]. This divergence might result from variations in pediatric age demographics and underlying health conditions, emphasizing the importance of context-specific RDW interpretation.

In alignment with existing literature, our study found elevated RDW levels correlated with prolonged LOS in the PICU. Patients with RDW greater than 14.4% had an average PICU stay of approximately five days, corroborating prior findings that elevated RDW independently predicts longer PICU stays [7, 8]. However, our results did not establish a statistically significant trend across RDW groups, indicating the need for further studies to define clear RDW thresholds for clinical decision-making.

Previous studies have consistently reported elevated RDW as an independent predictor of mortality among critically ill patients [5, 6]. Our study observed the highest mortality rate (approximately 14.9%) in patients with the highest RDW levels. Although this finding supports the trend observed in earlier studies, the association was not statistically significant, potentially due to limitations such as single-center data, retrospective design, and underlying hematological disorders not explicitly excluded beyond anemia and recent transfusions.

Sepsis, identified by elevated procalcitonin, was disproportionately represented among patients with varied RDW levels. Interestingly, patients with low RDW levels had higher sepsis rates, challenging previous literature that consistently associates elevated RDW with sepsis severity [16]. Elevated procalcitonin was notable among patients with both high and low RDW, suggesting that RDW’s predictive capability might vary significantly depending on underlying inflammatory responses, necessitating further investigation.

Contrary to expectations from previous studies, our study showed an inverse relationship between CRP levels and RDW, with higher CRP values observed in patients with lower RDW levels [6]. This paradoxical finding could be indicative of acute inflammatory episodes distinct from chronic inflammatory states that elevate RDW, warranting more detailed mechanistic studies.

Elevated RDW was associated with lower hemoglobin and mean corpuscular hemoglobin concentration (MCHC), supporting previous reports that elevated RDW reflects underlying anemia and ineffective erythropoiesis common in critical illness [3]. We mitigated bias by excluding patients diagnosed with anemia and recent transfusions, but residual confounding from undiagnosed hematological conditions could persist, as acknowledged in our limitations.

Additionally, our study explored the relationships between RDW and established pediatric severity scores such as PIM-2, PELOD-2, and PRISM III. Although slightly higher PIM-2 scores correlated with elevated RDW, consistent with previous findings, PRISM III scores showed limited association with RDW-related mortality, suggesting potential limitations of these scoring systems in capturing the prognostic implications of RDW [17].

The definition and early identification of MODS are critical in pediatric intensive care management. Although previous studies indicated RDW as a predictive biomarker for MODS, our findings did not establish a statistically significant trend [2]. Patients with moderate RDW elevations (13.4%−14.3%) showed a slightly higher incidence of MODS, but results were not statistically robust. Thus, our findings suggest RDW’s utility as an early marker for MODS requires further validation through larger prospective cohorts.

Our study has important limitations, including its retrospective, single-center design, potential selection and reporting biases, and inability to explicitly exclude all hematological disorders other than anemia and recent transfusions. Another limitation of our study was that we could not determine the precise day of MODS onset within the first 7 days of PICU stay. Consequently, while mean MODS values illustrate overall trends, they do not reflect the exact timing of onset. Additionally, some patients already had MODS at admission, which is common in critically ill cohorts and may influence the interpretation of temporal patterns. Future studies should address these limitations by employing prospective multicenter designs and more comprehensive exclusion criteria.

## Conclusion

In summary, despite not reaching statistical significance, our findings support a trend where elevated RDW levels could indicate increased mortality and prolonged LOS among pediatric patients in the PICU. While the study did not robustly associate RDW with early MODS prediction, it suggests the need for larger, prospective, multicenter studies to conclusively determine RDW thresholds’ clinical utility.

## Data Availability

Access to the electronic database was requested from the Pediatrics Department at Aga Khan University. The data is not made publicly available but it can be shared upon request via email to the corresponding author.

## Abbreviations

RDW: Red Cell Distribution Width
CBC: Complete Blood Count
ESR: Erythrocyte Sedimentation Rate
CRP: C-Reactive Protein
MODS: Multiple Organ Dysfunction Syndromes
MCV: Mean Corpuscular Volume
MCHC: Mean Corpuscular Hemoglobin Concentration
LOS: Length of Stay
PRISM III: Pediatric Risk of Mortality III
PICU: Pediatric Intensive Care Unit
BUN: Blood Urea Nitrogen
